# The effect of COVID-19 lockdown on the glycemic control of children with type 1 diabetes

**DOI:** 10.1186/s12887-022-03115-6

**Published:** 2022-01-19

**Authors:** Elina Hakonen, Tero Varimo, Anna-Kaisa Tuomaala, Päivi J. Miettinen, Mari-Anne Pulkkinen

**Affiliations:** Pediatric Research Center, New Children’s Hospital, Helsinki University Hospital, Stenbäckinkatu 9, HUS 00029, PO Box 347, Helsinki, Finland

**Keywords:** Type 1 diabetes, COVID-19 lockdown, Glycemic control, Continuous glucose monitoring, Children and adolescents

## Abstract

**Background:**

Between March 18^th^ and May 13^th^ 2020, the COVID-19 pandemic outbreak in Finland resulted in the closure of schools and the limitation of daycare (i.e. lockdown). Social distancing changed the daily routines of children with type 1 diabetes (T1D). Healthcare professionals were forced to adapt to the pandemic by replacing physical outpatient visits with virtual visits. However, the influence of the lockdown on glycemic control in these patients remained unknown.

**Methods:**

In this retrospective register study from a pediatric diabetes outpatient clinic, we analyzed the glycemic data of T1D patients (*n* = 245; aged 4 to 16 years) before and under the lockdown. All the participants used continuous glucose monitoring (rtCGM or iCGM), two-thirds were on insulin pumps (CSII), and one-third on multiple daily insulin injections (MDI) therapy.

**Results:**

In our patient cohort, time in range (TIR, *n* = 209) and mean glucose levels (*n* = 214) were similar prior to and under the lockdown (mean change 0.44% [95%CI: -1.1–2.0], *p* = 0.56 and -0.13 mmol/mol [95%CI: -0.3–0.1], *p* = 0.17, respectively). However, children treated with CSII improved their glycemic control significantly during the lockdown: TIR improved on average 2.4% [0.6–4.2] (*p* = 0.010) and mean blood glucose level decreased -0.3 mmol/mol [-0.6-(-0.1)] (*p* = 0.008). The difference was more pronounced in girls, adolescents and patients using conventional insulin pumps.

**Conclusions:**

The glycemic control in T1D children did not deteriorate under the lockdown, and patients on CSII even improved their control, which suggests that social distancing might have allowed families to use the insulin pump more accurately as out-of-home activities were on hold.

## Background

In March 2020, the outbreak of the novel coronavirus SARS-CoV-2 (COVID-19) led governments to take exceptional measures to counter the spread of the virus. Most countries implemented different degrees of lockdown to minimize the spread of the infection. In Finland, a state of emergency was declared on the 16^th^ of March. Primary school was arranged as homeschooling via video-teaching between March 18^th^ and May 13^th^ 2020. At the same time, remote working was encouraged, and outdoor team sports and leisure activities were suspended until June 1^st^ 2020. Daycare in kindergartens remained open during the lockdown in Finland, but parents were strongly encouraged to keep their children home whenever possible. The majority of parents followed the recommendation, and only approximately 30% of children participated in daycare during the lockdown [1].

Daily routines such as regular physical activities and psychosocial well-being have an important impact on glycemic control in children with type 1 diabetes (T1D) [2]. Daycare or primary school accounts for a significant part of the daily activities for all children. It has been shown that during the lockdown, the daily schedule of children changed dramatically [3, 4]. Evidence suggests, that the lockdown led to physical inactivity and an unhealthier diet [4], and 40% of adults with diabetes reported less exercise and increase in weight gain during the lockdown period [5]. Additionally, it has been hypothesized that the management of chronic diseases such as hypertension, mental diseases, and diabetes may have suffered due to limited access to outpatient clinics [6]. Few reports show some influence of the lockdown on the glycemic control of children with T1D. In study by Wu et al. children with T1D represented with lower number of hypoglycemia during the lockdown, but no change in TIR [7]. Di Dalmazi and colleagues reported that glycemic control, measured by continuous glucose monitoring (CGM), slightly improved (less time below range and smaller glucose SD) under lockdown in all children, except in teenagers with T1D [8]. In another study including 50 children and adolescents HbA1c was improved 0.2% (2 mmol/l) without increase in BMI during the lockdown [9], and a study by Marigliano and Maffeis with 233 children and adolescents also shows improved HbA1c, mean glucose and TIR during the lockdown [10], as does another study with 66 children from Piedmont, Italy [11]. Additionally, there are two studies on predictive low-glucose suspend system (PLGS) in children during COVID-19 with contradictory results: Schiaffini et al. showing that 22 children treated with PLGS improved their time – in – range (TIR) [12], whereas Christoforidis et al. showing with 34 children on PLGS system, that no difference in the glycemic control was observed during lockdown [3]. Furthermore, Tornese et al. demonstrated that glycemic control of T1DM adolescents using hybrid closed-loop (HCL) systems did not worsen during the COVID-19 pandemic and further improved in those who continued physical activity during the quarantine [13].

In this retrospective register study from a Finnish pediatric diabetes outpatient clinic, we evaluated the effect of the lockdown on glycemic control of T1D patients (*n* = 245; aged 4 to 16 years). We hypothesized that the lockdown with social distancing changed the daily routines of children with T1D and their families and resulted in deteriorating glycemic control. Furthermore, we analyzed whether the mode of treatment or age contributed to glycemic control under lockdown. To test this, we evaluated the changes in TIR and mean glucose sensor values in children with T1D prior to and under the lockdown.

## Methods

The study was conducted at the outpatient clinic of Jorvi Hospital, part of the hospital district of Helsinki and Uusimaa (HUS) and Helsinki University Hospital (HUH), which is responsible for the follow-up of 392 children (aged 1 – 16 years) with T1D. Normally, during each visit at the outpatient clinic, a diabetologist and a diabetes nurse meet the patient and the glycometric data (TIR, mean blood glucose and HbA1c, if available) is downloaded into the diabetes quality registry (BCB Medical). For the present study, the data from the BCB registry was retrieved for patients who had had a diabetes clinic appointment (virtual or live) between March 22^nd^ and May 20^th^ 2020 (lockdown). This glycemic data was compared to the data collected during the previous visit of the same patient (visit between November 1^st^ 2019 and March 21^st^ 2020, pre-lockdown period). Study inclusion criteria were age between 4 and 15.99 years, sufficient real-time continuous glucose monitoring (rtCGM) or integrated continuous glucose monitoring (iCGM) active data and at least two appointments at the diabetes outpatient clinic (one during lockdown and one pre-lockdown contact). Altogether, the data of 245 patients with T1D was retrospectively evaluated. The following data were retrieved for all patients: mean sensor glucose during and pre-lockdown, TIR and TBR (time below range) during and pre-lockdown, age, sex, year of diabetes diagnosis, treatment method (CSII or MDI), insulin daily dose per weight, insulin pump type (if applicable), sensor type, and pre-lockdown HbA1c.

TIR data were available in 209 patients and mean glucose levels in 214 patients. Patients were divided into three age groups: 4–6.9 years old (*n* = 20), 7–11.9 years old (*n* = 112), and 12–15.9 years old (*n* = 113). Additionally, patients were categorized based on their mode of treatment (CSII or MDI) and gender.

As this was a register-based study, the approval of an ethics committee was not required, but the research permit of the Helsinki University Hospital was obtained. The principles of Good Clinical Practice and the Declaration of Helsinki were followed and only the study personnel had access to the registry data handled.

### Statistical analyses

The data are presented with mean and 95% confidence intervals (CIs) unless otherwise stated. Analyses were performed with SPSS statistic for Windows (version 22.2, Chicago, IL). Between-group comparisons of the changes (i.e. variable under covid-19 lockdown minus before lockdown variable) in TIR and mean glucose level were performed with independent samples t-test (two groups) or with a one-way analysis of variance (ANOVA) (three groups). Within-group comparisons were analyzed with one sample t-test against zero. The level of statistical significance was set to p-value less than 0.05.

## Results

A total of 245 patients with T1D aged between 4 and 15.99 years were included in the analysis. Their mean age was 11 years (95% CI 10.7–11.4), and 130 (53%) patients were males. Of the patients, 80 (33%) were on multiple daily insulin injections (MDI), whereas 155 (63%) were on continuous subcutaneous insulin infusion (CSII) (Table 1). Ten subjects (4%) lacked information of the treatment method in the BCB registry (Table 1). TIR and TBR data were available from 209 patients (85%) and mean glucose levels from 214 patients (87%).

**Table 1:** Demographic and baseline characteristics of patients included in the study. Data are expressed as mean ± standard deviation (SD) or as number of patients (percentage). *TIR* time in range. *MDI* multiple daily injections

Parameter	Value
Number of patients	245
Male (n, %)	130 (53%)
Female (n, %)	115 (47%)
Age (years, mean ± SD)	11.06 ± 2.9
4–6.9 years (n)	20
7–11.9 years (n)	112
12–15.9 (n)	113
Disease durations (years, mean ± SD)	5.44 ± 3.3
Treatment	
Insulin pump (n, %)	155 (63%)
Omnipod	54 (35%)
Minimed 640G	26 (17%)
Minimed 670G	42 (27%)
Paradigm	31 (20%)
Accuchek combo	2 (1%)
MDI (n, %)	80 (33%)
Unknown (n, %)	10 (4%)
Glucosensor	
Freestyle libre	113 (46%)
Guardian	58 (24%)
Dexcom G6	36 (14.5%)
Unknown	38 (15.5%)
Prelockdown HbA1c (mmol/mol, mean ± SD)	58,9 ± 11.8 (*n* = 239)
TIR data available (n)	209
Mean sensor glucose available (n)	214

In the analysis of the total patient cohort, TIR and mean glucose levels were similar prior to and under the COVID-19 lockdown (mean change: 0.44% [95%CI: -1.1–2.0], *p* = 0.56 and -0.13 mmol/mol [95%CI: -0.3–0.1], *p* = 0.17, respectively). Neither gender nor age had an effect on TIR or mean glucose levels (*p* = 0.11–0.87). There was no significant change in TBR (5.9% pre-lockdown *vs.* 6.2% during lockdown, *p* = 0.44). Furthermore, no difference in the insulin total daily dose (TDD) (0.77 IU/kg/day pre-lockdown and during lockdown) could be detected. However, subjects on CSII therapy showed an improved TIR and lower mean glucose levels than those on MDI therapy (2.4% [0.6–4.2] *vs.* -2.6% [-5.2-(-0.19)], *p* = 0.002 and -0.3 [-0.6-(-0.1)] vs 0.2 [-0.1–0.5] mmol/mol, *p* = 0.013, respectively) with no change in TBR.

### Within group changes

**Fig. 1:**
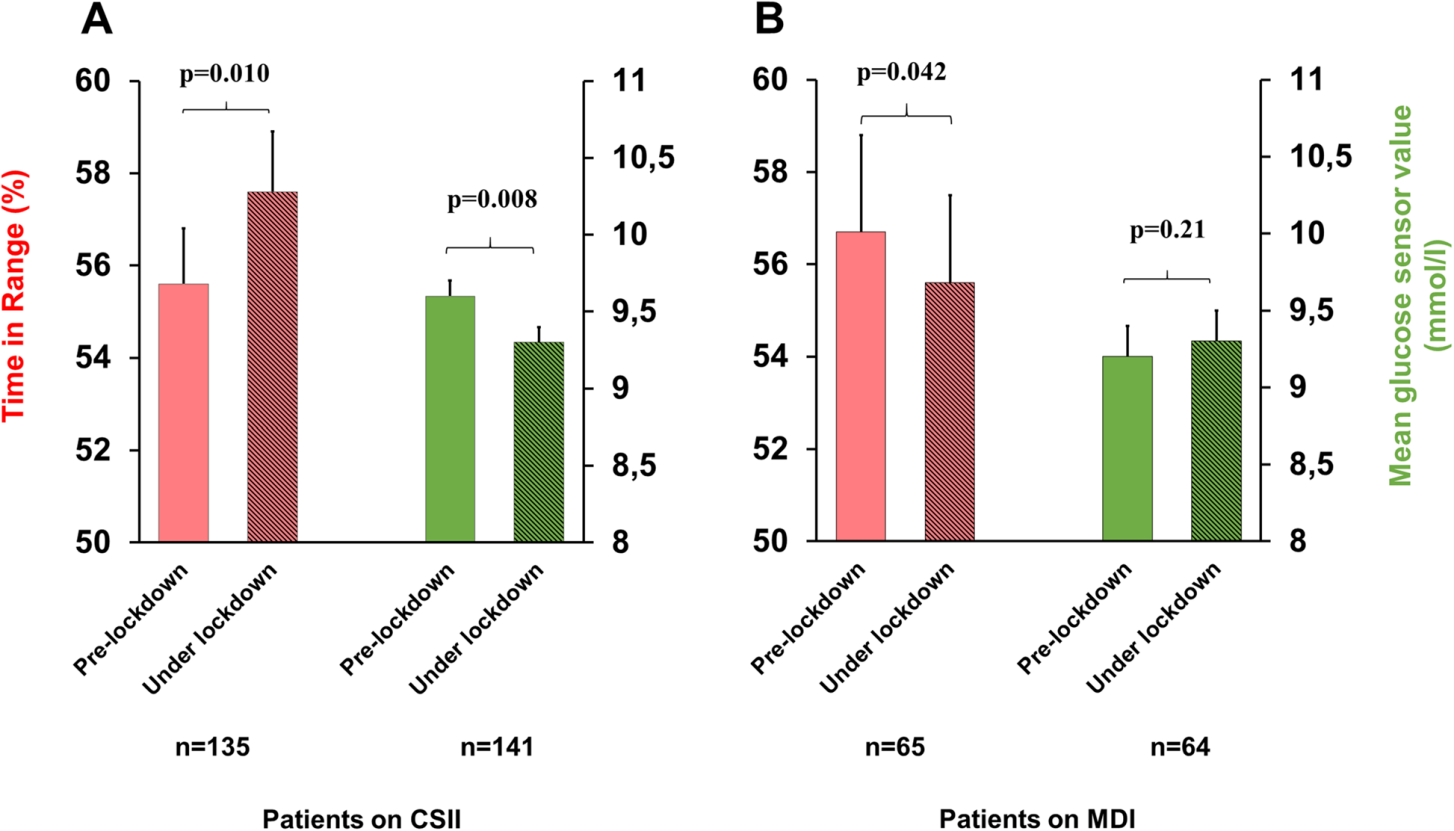
Time in Range (red) and mean sensor glucose value (green) in children and adolescent with T1DM on CSII (**A**), in patients on MDI (**B**) before and under COVID-19 lockdown. Mean (SEM)

In subjects on CSII, TIR improved under the lockdown period (mean 2.4 [0.6–4.2] %, *p* = 0.010) and mean glucose level decreased (-0.3 [-0.6-{-0.1}] mmol/mol, *p* = 0.008) (Fig. 1A), while TBR did not change (*p* = 0.86). Total daily dose of insulin remained the same before and during the lockdown (0.77 IU/kg/day vs 0.78 IU/kg/day, *p* = 0.77). A clear gender difference was evident as girls on CSII improved their TIR and lowered their mean sensor glucose level under the lockdown (3.9% [1.0–6.9], *p* = 0.010 and -0.5 [-0.9-{-0.1}] mmol/mol, *p* = 0.018), whereas similar improvements were not observed in boys (0.9% [-1.3–3.1], *p* = 0.41 and -0.2 [-0.4–0.1] mmol/mol, *p* = 0.22, respectively). At the same time, the oldest age group (i.e. 12–15.9 years) on CSII showed significant improvement in TIR (3.5% [0.2–6.8], *p* = 0.036), and a significant decrease in mean glucose level (-0.5 [-0.9-{-0.1}], *p* = 0.019).

**Fig. 2:**
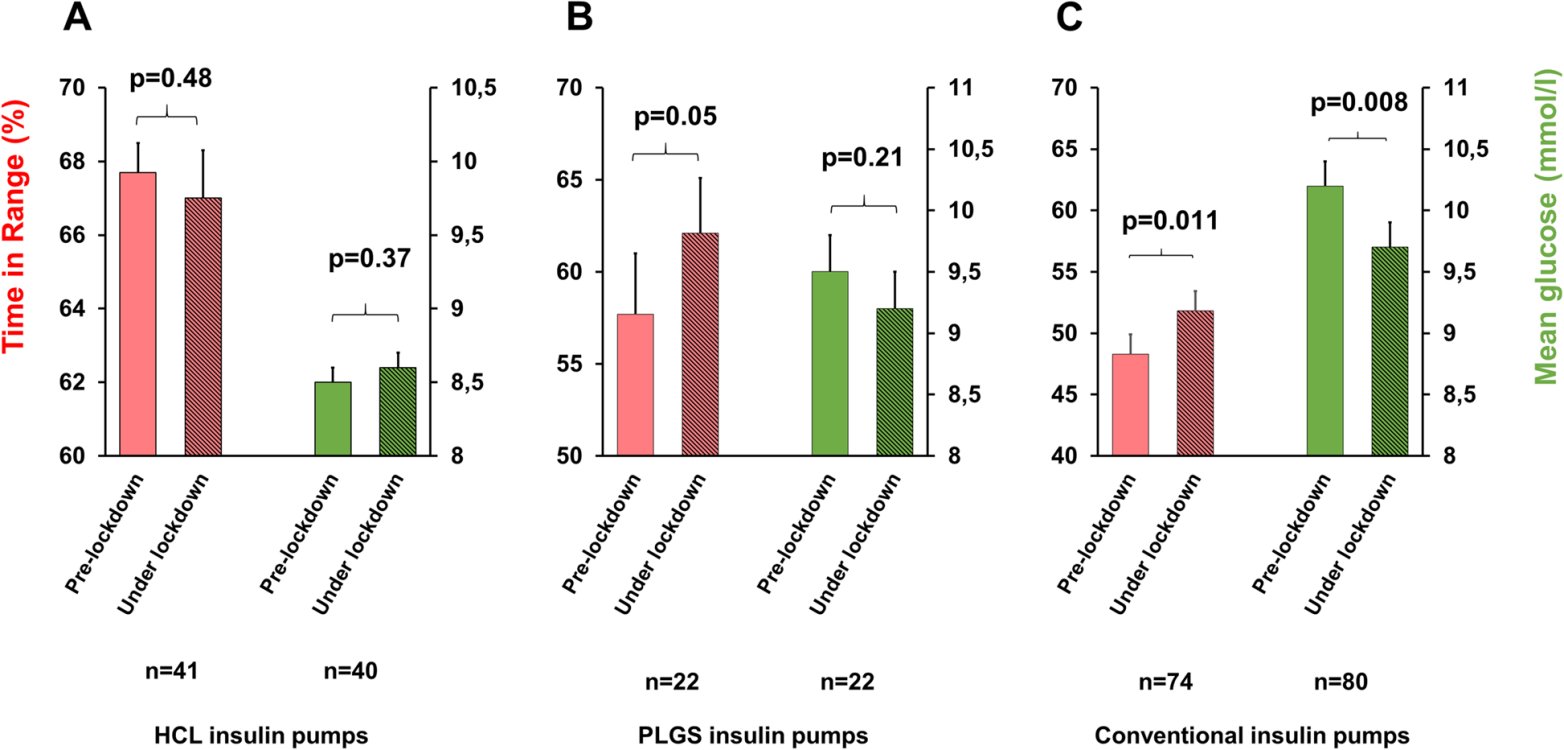
Time in Range (red) and mean sensor glucose value (green) in children and adolescent with T1DM on HCL (**A**), PLGS (**B**), and conventional insulin pumps  (**C**) before and under COVID-19 lockdown. Mean (SEM)

Surprisingly, subgroup analysis within the pumps types showed that patients using conventional (Omnipod, AccuCheck Combo, Paradigm) insulin pumps improved their TIR most (3.5% [0.8–6.1] mmol/mol, *p* = 0.011). Patients using Preditive Low Glucose Suspend ( PLGS) pumps had a trend for improvement in TIR (5% [0–10] mmol/mol, *p* = 0.05), while no change in TIR was observed in patients using HCL system (-0.9% [-3.4–1.6] mmol/mol, *p* = 0.48) (Fig. 2).

Notably, TIR decreased in patients on MDI under the lockdown, but no significant increase in mean sensor glucose level was observed (-2.6% [-5.2-{-0.1}], *p* = 0.042 and 0.2 [-0.1–0.5] mmol/mol, *p* = 0.21) (Fig. 1B). Similarly, TIR decreased significantly in the oldest age group on MDI (-3.9% [-7.9-{-0.1}], *p* = 0.047). Contrary to patients on CSII, no gender difference was seen in the MDI group (*p* = 0.05–0.8). Total daily dose of insulin did not change significantly (0.77 IU/kg/day pre-lockdown vs 0.74 IU/kg/day during lockdown, *p* = 0.82).

## Discussion

During the COVID-19 lockdown, daily routines were changed, possibly leading to reduced physical activity and increased screen and sleep time [4]. All these factors are known to have an impact on glycemic control in children and adolescents with T1D [14, 15]. We hypothesized that this could lead to worsening of glycemic control during the COVID-19 lockdown.

Surprisingly, this did not occur in any of the age groups or treatment subgroups. In fact, the glycemic control of T1D children on CSII improved without increased number of hypoglycemias. Our results are in line with recent reports on adult T1D patients working from home offices due to the COVID-19 lockdown, and reports on pediatric populations with improvement during the lockdown [9–11, 13, 16, 17]. Interestingly, in our study the improvement of glycemic control was most evident in the patients using conventional insulin pumps while no change was observed in those using HCL pumps. This suggests that having more time to focus on diabetes management, fine-tuning the pump, and having parents more involved in the daily diabetes treatment benefits especially those children treated with insulin pumps without hybrid closed loop –algorithm.

Patients on PLGS insulin pumps tended to have a slightly improved TIR during lockdown. To the best of our knowledge, there are two conflicting studies on the effects of CSII with PLGS in children during COVID-19 [3, 12]. Our results are in line with a recent study by Schiaffini et al. [12] but contrary to the already mentioned study by Christoforidis et al. on PLGS system [3]. It is noteworthy that in the study by Christoforidis et al., significant changes in daily schedule, including breakfast time later than normally in two-thirds of children, were reported during the lockdown [3]. In Finland, the remote school system was launched immediately after the implementation of a state of emergency. Since remote schools started similarly to pre-lockdown time, i.e. at 8 or 9 am, the daily rhythm of a normal school day was better maintained.

In our cohort, children on HCL pumps had a significantly better baseline glycaemia, possibly explaining why they did not improve their glycemic control during the lockdown. Interestingly, Tornese et al. demonstrated that glycemic control of T1DM adolescents using HCL systems did not worsen during COVID-19 pandemics and further improved in those patients who we able to continue their excercise during the quarantine [13]. Unfortunately, we were not able to analyze the patient’s physical activity due to the retrospective nature of our study. However, National Sports Council in Finland has showed that during the COVID-19 lockdown the school-aged children took 1000–3000 less steps per day when compared with the results on year 2018. This accounts approximately a 20% reduction in the amount of their exercise [18]. We assume that this applies also to our patient cohort.

Generally, adolescence is a challenging time for diabetes management [19]. Therefore, it was surprising that the oldest age (12–15.9 years) group using CSII improved their glycemic control significantly. During the holidays, the management of T1D is usually impaired especially in adolescents [20]. However, the lockdown differs from the holidays, as during the lockdown, teenagers were mostly at home instead of spending their leisure time with friends. Furthermore, teenagers’ daily rhythm was possibly more preserved due to remote school. We hypothesize that these two factors contribute to the improved glycemic control of the adolescents in the study. A study by Di Dalmazi et al. showed that glycemic control in a group of 24 teenagers did not deteriorate, nor did they see any improvement [8]. However, they did not analyze the adolescents on CSII as a separate group as we did. Also two other reports are in line with our finding: glycemic control tends to improve as adolescents stay home [10, 11], whereas another report of 102 T1D pediatric patients shows stable CGM metrics during a nationwide lockdown [21].

Finally, recent years have witnessed a revolution in the use of technology in the treatment and follow-up of T1D patients [22]. The COVID-19 pandemic enhanced the introduction of telemedicine in the management of diabetes [23, 24]. In our clinic, telemedicine was rapidly implemented at the beginning of the COVID-19 lockdown, since the professionals and most of the families already were familiar with CSII and CGM data downloading. Previously scheduled appointments were rapidly changed to virtual outpatient clinic visits. Thus, the intervals of the diabetes visits were not prolonged and the diabetes team was able to support and guide the patients and families at normal three-month visit intervals. Implementation of telemedicine appears to have a beneficial role in the maintenance of optimal glycemic control [24].

A strength of our study is the large patient cohort including patients on MDI and on CSII. In fact, to date this is the largest study on T1D children during the COVID-19 induced lockdown. A limitation of our study is the lack of information about changes in meals and physical activity before and during lockdown. It is also possible that some of the children, especially in the youngest age group, participated in daycare and were not at home during lockdown.

## Conclusions

This study showed that during COVID-19 lockdown, glycemic control of T1D children did not worsen despite the significant change in their lifestyle. Furthermore, limited access to outpatient clinic and transfer to virtual visits did not deteriorate their glycemic control. Instead, children on CSII improved TIR and mean blood glucose during the lockdown without increase in TBR and the difference was even more marked in adolescents, girls and patients using conventional insulin pumps. Social distancing forced families together and made the family environment more cohesive, which may have improved glycemic control of T1D children on CSII.

## Data Availability

The datasets used and analy*z*ed during the current study are available from the corresponding author on reasonable request.

## Abbreviations

CGM: Continuous glucose monitoring
CSII: Insulin pump / continuous subcutaneous insulin infusion
HCL: Hybrid closed-loop
MDI: Multiple daily insulin injections
iCGM: Integrated continuous glucose monitoring
PLGS: Predictive low glucose suspend
rtCGM: Real-time continuous glucose monitoring
TBR: Time below range
TIR: Time in range
T1D: Type 1 diabetes

## References

[1] https://avi.fi/tiedote/-/tiedote/69887908. Accessed 17 May 2021

[2] American Diabetes Association (2019). 13. Children and Adolescents: Standards of Medical Care in Diabetes-2019. Diabetes Care.

[3] Christoforidis A, Kavoura E, Nemtsa A (2020). Coronavirus lockdown effect on type 1 diabetes management οn children wearing insulin pump equipped with continuous glucose monitoring system. Diabetes Res Clin Pract.

[4] Pietrobelli A, Pecoraro L, Ferruzzi A (2020). Effects of COVID-19 Lockdown on lifestyle behaviors in children with obesity living in Verona, Italy: A Longitudinal Study. Obesity (Silver Spring).

[5] Ruissen MM, Regeer H, Landstra CP (2021). Increased stress, weight gain and less exercise in relation to glycemic control in people with type 1 and type 2 diabetes during COVID-19 pandemic. BMJ Open Diabetes Res Care.

[6] Saqib MAN, Siddiqui S, Qasim M (2020). Effect of COVID-19 lockdown on patients with chronic diseases. Diabetes Metab Syndr.

[7] Wu X, Luo S, Zheng X (2021). Glycemic control in children and teenagers with type 1 diabetes around lockdown for COVID-19: A continuous glucose monitoring-based observational study. J Diabetes Investig.

[8] Di Dalmazi G, Maltoni G, Bongiorno C (2020). Comparison of the effects of lockdown due to COVID-19 on glucose patterns among children, adolescents, and adults with type 1 diabetes: CGM study. BMJ Open Diabetes Res Care.

[9] Cognigni M, D'Agostin M, Schiulaz I (2021). HbA1c and BMI after lockdown for COVID-19 in children and adolescents with type 1 diabetes mellitus. Acta Paediatr.

[10] Marigliano M, Maffeis C (2021). Glycemic control of children and adolescents with type 1 diabetes improves after COVID-19 lockdown in Italy. Acta Diabetlogica.

[11] Tinti D, Savastio S, Grosso C (2021). Impact of lockdown during COVID-19 emergency on glucose metrics of children and adolescents with type 1 diabetes in Piedmont. Italy Acta Diabetol.

[12] Schiaffini R, Barbetti F, Rapini N (2020). School and pre-school children with type 1 diabetes during Covid-19 quarantine: The synergic effect of parental care and technology. Diabetes Res Clin Pract.

[13] Tornese G, Ceconi V, Monasta L (2020). Glycemic Control in Type 1 Diabetes Mellitus During COVID-19 Quarantine and the Role of In-Home Physical Activity. Diabetes Technol Ther.

[14] Miculis CP, De Campos W, da Silva Boguszweski MC (2015). Correlation between glycemic control and physical activity level in adolescents and children with type 1 diabetes. J Phys Act Health.

[15] Kelo M, Martikainen M, Eriksson E (2011). Self-care of school-age children with diabetes: an integrative review. J Adv Nurs.

[16] Fernández E, Cortazar A, Bellido V (2020). (2020) Impact of COVID-19 lockdown on glycemic control in patients with type 1 diabetes. Diabetes Res Clin Pract.

[17] Bonora BM, Boscari F, Avogaro A (2020). Glycaemic Control Among People with Type 1 Diabetes During Lockdown for the SARS-CoV-2 Outbreak in Italy. Diabetes Ther.

[18] https://www.liikuntaneuvosto.fi/wp-content/uploads/2020/10/Koronapandemian-vaikutukset-vaeston-liikuntaan-paivitetty-23.11.2020.pdf

[19] Rausch JR, Hood KK, Delamater A (2012). Changes in treatment adherence and glycemic control during the transition to adolescence in type 1 diabetes. Diabetes Care.

[20] Landau Z, Lebenthal Y, Boaz M, Pinhas-Hamiel O (2013). Observational study of diabetes management in type 1 diabetic school-age children during holiday versus school days. J Pediatr Endocrinol Metab.

[21] Brener A, Mazor-Aronovitch K, Rachmiel M (2020). Lessons learned from the continuous glucose monitoring metrics in pediatric patients with type 1 diabetes under COVID-19 lockdown. Acta Diabetol.

[22] van den Boom L, Karges B, Auzanneau M (2019). Temporal Trends and Contemporary Use of Insulin Pump Therapy and Glucose Monitoring Among Children, Adolescents, and Adults With Type 1 Diabetes Between 1995 and 2017. Diabetes Care.

[23] Tornese G, Schiaffini R, Mozzillo E (2021). Telemedicine in the Time of the COVID-19 Pandemic: Results from the First Survey among Italian Pediatric Diabetes Centers. Healthcare (Basel, Switzerland).

[24] Predieri B, Leo F, Candia F (2020). Glycemic Control Improvement in Italian Children and Adolescents With Type 1 Diabetes Followed Through Telemedicine During Lockdown Due to the COVID-19 Pandemic. Front Endocrinol.

